# Human Parvovirus 4 as Potential Cause of Encephalitis in Children, India

**DOI:** 10.3201/eid1708.110165

**Published:** 2011-08

**Authors:** Laura A. Benjamin, Penny Lewthwaite, Ravi Vasanthapuram, Guoyan Zhao, Colin Sharp, Peter Simmonds, David Wang, Tom Solomon

**Affiliations:** Author affiliations: University of Liverpool Institute of Infection and Global Health, Liverpool, UK (L.A. Benjamin, P. Lewthwaite, T. Solomon);; Walton Centre National Health Service Foundation Trust, Liverpool (L.A. Benjamin, T. Solomon);; Vijayanagar Institute of Medical Sciences, Bellary, India (R. Vasanthapuram);; Washington University, St. Louis, Missouri, USA (G. Zhao, D. Wang);; University of Edinburgh, Edinburgh, Scotland (C. Sharp, P. Simmonds)

**Keywords:** viruses, human parvovirus, high throughput sequencing, PARV4, encephalitis, epidemiology, pediatrics, children, India, dispatch

## Abstract

To investigate whether uncharacterized infectious agents were associated with neurologic disease, we analyzed cerebrospinal fluid specimens from 12 children with acute central nervous system infection. A high-throughput pyrosequencing screen detected human parvovirus 4 DNA in cerebrospinal fluid of 2 children with encephalitis of unknown etiology.

Encephalitis is a major cause of death and disability globally. In Asia, Japanese encephalitis virus is the most commonly recognized cause, with 30,000–50,000 cases and ≈10,000 deaths annually (*1*). However, for most encephalitis cases, the cause is unknown, even with comprehensive screening for recognized agents (*1*).

The advent of high-throughput sequencing technologies enabled the direct characterization of microbial nucleic acid from clinical samples and revolutionized the process for identifying potential etiologic agents of disease. A key feature is that the method is unbiased and can detect any nonhost nucleic acid sequences present in a sample. It has the potential to detect expected agents, known but unexpected agents, and novel agents (*2*).

Human parvovirus 4 (PARV4) is a single-stranded DNA virus first detected in 2005 (*3*). Infections with PARV4 are accompanied by acute viremia for several weeks (C. Sharp et al., unpub. data), followed by seroconversion for antibody and virus clearance. As observed with another human parvovirus, parvovirus B19, there is long term persistence of viral DNA sequences in several tissues but not the brain (*4*). We describe 2 children in southern India with suspected encephalitis and high PARV4 levels in their cerebrospinal fluid (CSF).

## The Study

We obtained CSF from a cohort of children (<16 years of age) hospitalized with a suspected acute central nervous system (CNS) infection at the Vijayanagar Institute of Medical Sciences, Bellary, India, October 2005–October 2007, as previously described (*5*). Suspected CNS infection was defined as a febrile illness (for <2 weeks) and >1 of the following signs or symptoms: severe headache, altered mental status, seizures, or focal neurologic signs (*6*).

To investigate whether uncharacterized infectious agents were associated with neurologic disease, we obtained CSF specimens from 12 patients with acute CNS infection (i.e., febrile illness with CSF leukocyte count >5 cells/mm^3^ or protein >45 mg/dL). These patients had negative diagnostic test results for pathogens known to cause CNS infection in this region of India at the time of investigation (e.g., Japanese encephalitis virus, chikungunya virus, dengue fever virus, and *Plasmodium falciparum*); in addition, CSF culture was performed and no bacterial organisms were found (*5*). Total nucleic acid was extracted from whole CSF and randomly amplified as previously described with the modification that 6-nt barcodes were added to the 5′ end of the primers used for the amplification (*7*). The amplified materials were pooled together and processed by using a high-throughput pyrosequencing technique on a GS FLX Titanium Platform (454 Life Sciences/Roche, Branford, CT, USA).

The raw sequence reads were deconvoluted on the basis of the barcode and then processed through a standardized bioinformatic pipeline (*2*). The sequences of interest were then categorized into taxonomy groups based on the best BLAST (www.ncbi.nlm.nih.gov/BLAST/) hit. For 2 of the 12 patients, patients VES085 and VES065, viral sequences were detected in the CSF.

We identified 17 (92.6%–98.2% sequence identity) distinct sequence reads in the CSF of patient VES085 and 6 (95.6%–98.9%, sequence identity) distinct reads from patient VES065 with pairwise identities based on BLASTn alignment of each read to the reference genome (GenBank accession no. EU175855.1). To verify the results, we used PARV4-specific PCR primers to screen all 12 original CSF samples as described (*8*). Only samples from VES085 and VES065 were positive; all other samples were negative by PCR.

PARV4 viral loads in the 2 CSF samples and the corresponding serum sample from 1 patient collected contemporaneously were semiquantified by limiting dilution PCR by using primers on 5 replicate samples of each dilution. The PCR conditions demonstrated single copy sensitivity (data not shown), and endpoint titers of 50% positivity were calculated by using the Reed-Muench formula (*8**,**9*). Both the CSF and 1 serum sample demonstrated high endpoint titers, indicating acute infection and substantial virus spread into the CNS of the 2 patients (Table 1).

**Table 1:** Clinical and laboratory characteristics of children with CSF positive for human parvovirus 4, India*

ID, date of illness	Age, y	Clinical course	Outcome	CSF					Serum		
PCR	WCC†	Protein, mg/dL	Glucose, mg/dL	IgM	IgG	PCR
VES085, 2006 Jan	2	Prodomal illness for 12 days; febrile with frequent generalized convulsions during first week of admission; CSF examination on day 12 of illness	Discharged against medical advice on day 18 after admission	+ 1.5 x 10^7^ copies/mL	4	59	32		+	−	+ 5.6 x 10^9^ copies/mL
VES065 2005 Nov	3	Prodomal illness for 9 days; febrile, poor appetite, and a generalized convulsion on day 9; CSF examination on day 9 of illness	Recovered on day 14 with no residual neurologic deficit	+ 3.2 x 10^5^ copies/mL	8	15	60		NA	NA	NA

*CSF, cerebrospinal fluid; ID, patient identification code; WCC, leukocyte count; Ig, immunoglobulin; +, positive; −, negative; NA, not available. †Leukocyte count differential 100% lymphocytes for each patient.

To genetically characterize PARV4 variants infecting the patients, we generated overlapping sets of amplicons spanning the complete genome of PARV4 from the 3 samples. No variation was seen between the CSF and serum-derived sequences from VES085. The fully assembled coding regions of the variants generated from both patients were compared to previously described PARV4 variants (n = 18 viral proteins 2/1 and n = 19 nonstructural sequences) (Table 2). Phylogenetic analysis demonstrated that both variants belonged to genotype 2 (Figure). All sequences have been submitted to GenBank (accession nos. HQ593530–HQ593532).

**Table 2:** Percent divergence of the nonstructural coding region of human PARV4–positive encephalitis patients VES065 and VES085, compared with previously described PARV4 variants*

Patient or genotype	Genotype 3, aa (nt), n = 1	Genotype 2, aa (nt), n = 7	Genotype 1, aa (nt), n = 11	VES085 serum, aa (nt), n = 1	VES085 CSF, aa (nt), n = 1	VES065 CSF, aa (nt), n = 1	
VES065 CSF, n = 1	9.5 (3.5)	3.7 (1.5)	10.3 (3.7)	0.2 (0.0)	0.2 (0.0)		
VES085 CSF, n = 1	9.4 (3.5)	3.6 (1.5)	10.2 (3.7)	0.0 (0.0)			
VES085 serum, n = 1	9.4 (3.5)	3.6 (1.5)	10.2 (3.7)				
Genotype 1, n = 11	8.0 (2.6)	9.3 (2.9)	0.9 (0.6)				
Genotype 2, n = 7	8.6 (2.7)	1.8 (0.7)					
Genotype 3, n = 1							

*PARV4, human parvovirus 4; CSF, cerebrospinal fluid.

**Figure:**
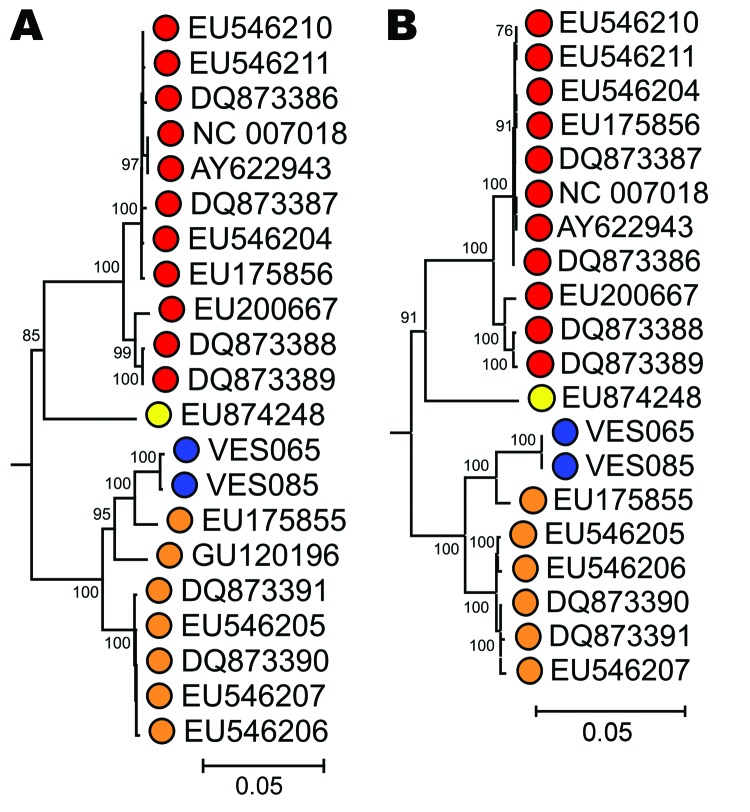
Phylogenetic analysis of (A) complete nonstructural (NS) and (B) viral protein (VP) 1/VP 2 gene sequences of human parvovirus 4 (PARV4) variants isolated from patients with encephalitis of unknown etiology using 167 available sequences from human PARV4 variants (genotypes 1–3). Blue, study sample; red, genotype 1; orange, genotype 2; yellow, genotype 3. The porcine hokovirus sequence (GenBank accession no. EU200671) was used as an outgroup (not shown). The trees were constructed by neighbor-joining of pairwise maximum-composite likelihood corrected distances between nucleotide sequences; bootstrap values ≈70% are shown. Scale bars represent an evolutionary distance of 0.05.

The serum sample was screened for immunoglobulin (Ig) G and IgM PARV4-specific antibodies by using a previously developed serologic assay for PARV4 antibodies (*10*). The serum of VES085 was IgM positive and IgG negative, consistent with an acute infection (Table 1).

Through serologic screening of age-matched controls, we were able to largely rule out the possibility that detection of PARV4 DNA in samples from 2 of the 12 patients was simply incidental to a high background incidence of PARV4 infection in this age group. Although published data on the duration of viremia in patients with acute PARV4 infections has not been directly determined, viral loads as found in the study patients are of relatively short duration in other parvovirus infections (16 days for viral loads >5,000 DNA copies/mL in the case of B19) (*11*). Furthermore, through testing sequential samples from persons exposed to PARV4, we have found no persons with viremia duration of >6 weeks (C. Sharp et al., unpub. data). Using the 6-week (maximum) estimate and a background incidence of infection of 2.2% per year (based on the detection of 4/41 seropositive children with a mean age of 4.5 years), the likelihood of viremia detection would be at most 0.25%, a figure consistent with the actual measured absence of viremia detection in the control cohort.

## Conclusions

PARV4 is a recently identified virus found in human blood and in a variety of tissues but with no known disease association. In this study we detected PARV4 DNA in 2 of 12 CSF samples from patients with suspected encephalitis of unknown cause in southern India. Unfortunately, no samples were available from the wider cohort for further testing. The presence of PARV4 DNA and IgM against PARV4 in acute-phase serum from the 1 child with this sample available further supports the contention that this was an acute infection.

Brain biopsy, the definitive investigation, was not available at the time. More often than not, clinicians have to rely on surrogate markers to diagnose encephalitis (e.g., CSF pleocytosis, febrile illness, and focal neurologic signs in the right clinical context). It is more common to have CSF pleocytosis than not, though of course there are well characterized cases of CNS infection (e.g., dengue) with no pleocytosis (*6*).

CSF erythrocyte count was not routinely measured in this resource-limited setting. Although it is theoretically possible the parvovirus detected in the CSF could have reflected spillover from a blood-contaminated spinal tap, there was no overt evidence of hemorrhagic CSF. The fact that patient VES085 was febrile with a neurologic syndrome and positive serum IgM, and patient VES065 had similar clinical signs with high PARV4 viral CSF levels suggests that they did indeed have a CNS infection.

PARV4 is primarily found in injection drug users and persons who have received blood products; PARV4 is therefore presumed to be transmitted parenterally. However, the risk for such transmission in the children in our study was low, which supports the possibility of an alternative route of transmission (*8**,**12*). HIV testing was not available.

The use of high-throughput sequencing for identifying an unexpected possible cause of CNS infection (PARV4) is cutting edge; this technique’s utility also extends to a recent identification of astrovirus in a case of encephalitis (*13*). With the high seroprevalence of PARV4 infection highlighted in recent reports (5%–37%), there is a clear need to further assess the role of PARV4 in CNS infection across the globe (*14**,**15*).
